# Dataset of characteristic remanent magnetization and magnetic properties of early Pliocene sediments from IODP Site U1467 (Maldives platform)

**DOI:** 10.1016/j.dib.2019.104666

**Published:** 2019-10-15

**Authors:** Luca Lanci, Elena Zanella, Luigi Jovane, Simone Galeotti, Montserrat Alonso-García, Carlos A. Alvarez-Zarikian, Nagender Nath Bejugam, Christian Betzler, Or M. Bialik, Clara L. Blättler, Gregor P. Eberli, Junhua Adam Guo, Sébastien Haffen, Senay Horozal, Mayuri Inoue, Dick Kroon, Juan Carlos Laya, Anna Ling Hui Mee, Thomas Lüdmann, Masatoshi Nakakuni, Kaoru Niino, Loren M. Petruny, Santi D. Pratiwi, John J.G. Reijmer, Jesús Reolid, Angela L. Slagle, Craig R. Sloss, Xiang Su, Peter K. Swart, James D. Wright, Zhengquan Yao, Jeremy R. Young

**Affiliations:** a Department of Pure and Applied Science, University of Urbino, Via S. Chiara 27, 61029 Urbino, Italy; b Alpine Laboratory of Paleomagnetism ALP - CIMaN, Via G.U. Massa 6, 12016 Peveragno, Italy; c Department of Earth Sciences, University of Turin, Via Valperga Caluso 35, 10125 Turin, Italy; d Instituto Oceanográfico da Universidade de São Paulo, Praça do Oceanográfico, 191, São Paulo, SP 05508-120, Brazil; e Divisão de Geologia e Georecursos Marinhos, Instituto Portugues do Mar e da Atmosfera (IPMA), Avenida de Brasilia 6, 1449-006 Lisbon, Portugal; f Centro de Ciencias do Mar (CCMAR), Universidade do Algarve, Faro, Portugal; g International Ocean Discovery Program, Texas A&M University, 1000 Discovery Drive, College Station, TX 77845, USA; h Geological Oceanography Division, CSIR-National Institute of Oceanography, Dona Paula, Goa 403004, India; i Institute for Geology, CEN, University of Hamburg, Bundesstrasse 55, 20146 Hamburg, Germany; j Dr. Moses Strauss Department of Marine Geosciences, The Leon H. Charney School of Marine Sciences, University of Haifa, 31905 Carmel, Israel; k Department of the Geophysical Sciences, University of Chicago, 5734 S. Ellis Ave., Chicago, IL 60637, USA; l Department of Marine Geosciences, Department of Marine Geosciences, Rosenstiel School of Marine and Atmospheric Science, University of Miami, Miami, FL 33149, USA; m Department of Geological Sciences, California State University Bakersfield, 9001 Stockdale Highway, Bakersfield, CA 93311, USA; n Physical Properties Specialist, Ecole Nationale Superieure de Geologie, Universite de Lorraine, 2 rue du Doyen Marcel Roubault, 54501 Vandoeuvre-les-Nancy, France; o Petroleum and Marine Research Division, Korea Institute of Geoscience and Mineral Resources (KIGAM), Gwahang-no 124, Yuseong-gu, Daejeon, 305-350, South Korea; p Graduate School of Natural Science and Technology, Okayama University, 3-1-1 Tsushima-naka, Okayama 700-8530, Japan; q Department of Geology and Geophysics, University of Edinburgh, Grant Institute, The King's Buildings, West Mains Road, Edinburgh EH9 3JW, UK; r Department of Geology and Geophysics, Texas A&M University, Mail Stop 3115, College Station, TX 77843-3115, USA; s Department of Environmental Engineering for Symbiosis, Soka University, 1-236 Tangi-cyo, Hachioji-shi, Tokyo 192-0003, Japan; t Graduate School of Science and Engineering, Yamagata University, 1-4-12 Kojirakawa-machi, Yamagata City 990-8560, Japan; u Environmental Science and Policy Department, George Mason University, David King Hall Rm 3005, MSN 5F2, 4400 University Drive, Fairfax, VA 22030-4444, USA; v Department of Geosciences, Geological Engineering Faculty, Universitas Padjadjaran, Jl.Raya Bandung Sumedang Km.21v, Jatinangor, 45363, Indonesia; w College of Petroleum Engineering and Geosciences, King Fahd University of Petroleum and Minerals, Dhahran 31261, Saudi Arabia; x Departamento de Estratigrafía y Paleontología, Universidad de Granada, Avenida de La Fuente Nueva S/N, 18071, Granada, Spain; y Lamont-Doherty Earth Observatory, Columbia University, Borehole Bldg. 61 Route 9W, Palisades, NY 10964, USA; z Earth and Environmental Sciences, University of Technology Queensland, R-Block 317, 2 George Street, Brisbane, QLD 4001, Australia; aa Key Laboratory of Marginal Sea Geology, South China Sea Institute of Oceanology, Chinese Academy of Sciences, 164 West Xingang Road, Guangzhou, 510301, People's Republic of China; ab Department of Geological Sciences, Rutgers, The State University of New Jersey, 610 Taylor Road, Piscataway, NJ, 08854-8066, USA; ac Department of Marine Geology, First Institute of Oceanography (FIO) State Oceanic Administration (SOA), #6 Xian Xia Ling Road, Qingdao, 266061, Shandong Province, People's Republic of China; ad Laboratory for Marine Geology, Qingdao National Laboratory for Marine Science and Technology, Qingdao, People's Republic of China; ae Department of Earth Sciences, University College London, Gower Street, London, WC1E 6BT, UK

**Keywords:** Paleomagnetism, Pliocene magnetic stratigraphy, Anisotropy of isothermal remanent magnetization, Currents strength, Monsoon

## Abstract

This data article describes data of magnetic stratigraphy and anisotropy of isothermal remanent magnetization (AIRM) from “Magnetic properties of early Pliocene sediments from IODP Site U1467 (Maldives platform) reveal changes in the monsoon system” [1]. Acquisition of isothermal magnetization on pilot samples and anisotropy of isothermal remanent magnetization are reported as raw data; magnetostratigraphic data are reported as characteristic magnetization (ChRM).

**Table:** Specifications Table

Subject	Earth and Planetary Sciences (General)
Specific subject area	Paleomagnetism; Rock-magnetism; Anisotropy of Isothermal Remanent Magnetization
Type of data	Excel file
How data were acquired	Natural Remanent Magnetization (NRM) and Isothermal Remanent Magnetization acquisition were measured with a 2G-enterprise DC-SQUIDs cryogenic magnetometer and stepwise demagnetized with on-line 2G AF demagnetizer. AIRM was measured with AGICO JRS-6 Spinner magnetometer.
Data format	Mixed (raw and analysed)
Parameters for data collection	The characteristic remanent magnetization were calculated from the AF-demagnetized NRM using the method of the principal component analysis [2] and the PuffinPlot software [3].
Description of data collection	Standard paleomagnetic cubes with a volume of 7 cm^3^ collected in Site U1467 from core sections 359-U1467B-11H to 359-U1467B-34H and from 359-U1467C-10H to 359-U1467C-17H
Data source location	Maldives platform, Indian Ocean (4° 51.0155′ N and 73° 17.0204′ E).
Data accessibility	With the article.
Related research article	Lanci et al. (2019), Magnetic properties of early Pliocene sediments from IODP Site U1467 (Maldives platform) reveal changes in the monsoon system. *Palaeogeography Palaeoclimatology Palaeoecology*, 533, 109283, https://doi.org/10.1016/j.palaeo.2019.109283.

**Table:** 

**Value of the Data** • Raw and analysed dataset present anisotropy of isothermal remanent magnetization (IRM) of sediments from IODP Site U1467 dated with geomagnetic polarity reversal sequence, which support the interpretation of the related research article [1] and could be useful to other researchers to understand the paleo-oceanography and the monsoon dynamics during the early Pliocene. • For future investigations bottom current strength and direction inferred from anisotropy of IRM, can provide clues on paleo-monsoon strength and their time variability. • Magnetostratigraphic age model could provide a starting point to develop a high-resolution astrochronological age model of Site U1467; anisotropy of IRM could be extend toward other IODP Sites and provide a more complete picture of paleo-monsoon strength.

## 1 Data

This dataset describes the acquisition of IRM, the median destructive field, the magnetostratigraphy and the anisotropy of IRM of the Pliocene sediments from IODP Site U1467.

IRM acquisition and the median destructive field describe the magnetic mineralogy of the sediments (see Ref. [1], Figure 2). Magnetostratigraphic data describes the direction of geomagnetic pole and are reported as characteristic remanent magnetization (ChRM) and as latitude of virtual geomagnetic pole (see Ref. [1] Figure 5). The intensity of natural remanent magnetization, the number of demagnetization steps and the maximum angular deviation are included to access the quality of the data. The age model data describe the magnetostratigraphic age of the sediments (see Ref. [1] Figure 6).

Anisotropy data describes the statistical orientation of elongated magnetic particles. They are reported as magnitude and direction of the main anisotropy axis (I_1_, I_2_, and I_3_). The descriptive parameters P′ and T (see Ref. [1], Figure 4) are also reported for practical purpose although they can be calculated from the anisotropy axis.

Data are reported in a table format as Excel data sheet. Values are described in Table 1.

**Table 1:** Variables description.

Variable	Type	Description
ID	Categorical	Specimen identification reported as Hole, Core type and number, Core section and Top section offset.
Depth	Numeric	Specimen depth in meter CSF-A.
NRM_moment	Numeric	Magnetic moment of natural remanent magnetization.
Demag_Steps	Integer	Number of alternating field demagnetization steps.
PCA_dec	Numeric	Declination of ChRM.
PCA_inc	Numeric	Inclination of ChRM.
PCA_MAD	Numeric	Maximum angular deviation of ChRM.
PCA_anchored	Categorical	Indicate if ChRM was computed as anchored to the origin (Y) or not anchored (N).
VGP_lat	Numeric	Latitude of virtual geomagnetic pole computed from the entire set.
IRM	Numeric	Intensity of IRM
P_prime	Numeric	Corrected anisotropy degree.
T	Numeric	Shape parameter of anisotropy ellipsoid.
I	Numeric	Normalized magnitude of anisotropy axes.
I_dec	Numeric	Declination of anisotropy axes.
I_inc	Numeric	Inclination of anisotropy axes.
flow_direction	Numeric	Inferred azimuthal direction of paleocurrents.
Field	Numeric	IRM acquisition field.
MDF	Numeric	Median destructive field of NRM.
Age	Numeric	Age of biostratigraphic events and magneto-chrons.
Datum	Categorical	Indicates the kind of chronological event.
Chron/Event	Categorical	Indicate the specific chronological event.

## 2 Experimental design, materials, and methods

Standard paleomagnetic specimens (plastic cubes, with a volume of 7 cm^3^) were collected in the upper part of Site U1467 from to 84 m–302 m core depth below sea floor (CSF-A). Specimens were collected from azimuthally-oriented APC cores.

IRM was acquired in a set of pilot specimens with 12 stepwise increasing fields from 0.03 T to 1 T, induced using a ASC pulse magnetizer and measured with a 2G-Enterprise, superconducting DC-SQUID magnetometer.

Natural remanent magnetization was measured using a 2G-enterprise DC-SQUID magnetometer at the and progressively demagnetized in alternating field up to the maximum field of 100 mT according to a standard paleomagnetic procedure. Characteristic magnetization were calculated using the principal component analysis [2] and the PuffinPlot software [3]. Median destructive field was computed from the alternating field demagnetization of NRM.

Anisotropy of IRM was induced in a field of 20 mT. IRM was measured and then AF demagnetized using a tumbling 2G AF-demagnetizer at a maximum field of 80 mT, before inducing the magnetization in the next axes. IRM was induced along 6 different axes and each axis was measured twice along opposite directions for a total of 12 measurements in each specimen. The intensity IRM was measured with a JR-6 spinner magnetometer. The anisotropy tensor and the directions of the principal IRM axis I_i_ (i.e., the eigenvectors of the AIRM tensor) were computed from the remanent magnetization using the AGICO software Anisoft42.

Flow directions is inferred after recognising the pattern of each specimen by comparing the angle q between the direction of the magnetic lineation I_1_ and the plunge of foliation plane. If the q < 35° the pattern is considered flow-aligned and the flow is taken equal to declination of the I_1_ axis in the direction of the foliation imbrication. If q ≥ 55° the pattern is consider flow-transverse and the flow is the declination of I_1_ – 90° in the direction of the foliation imbrication. The intermediate case (35°< q ≤ 55°) is handled by taking directly the imbrication direction of the foliation plane as the flow direction.
